# Emulating the LEADER trial in China: a regulatory science case study on non-interventional research

**DOI:** 10.3389/fendo.2026.1777954

**Published:** 2026-03-12

**Authors:** Jun Zhao, Xiaona Xin, Yuanyuan Song, Jie Zhang, Ying Wu, Jun Wang, Fuqiang Cui

**Affiliations:** 1 Department of Epidemiology and Biostatistics, School of Public Health, Peking University, Beijing, China; 2 Department of Statistics and Clinical Pharmacology, Center for Drug Evaluation, National Medical Products Administration, Beijing, China; 3 Department of Clinical Evaluation 1, Center for Drug Evaluation, National Medical Products Administration, Beijing, China; 4 Department of Biostatistics, Southern Medical University, Guangzhou, Guangdong, China; 5 Hainan Lecheng Institute of Real World Study, Qionghai, Hainan, China; 6 Peking University Medical and Health Analysis Center, Peking University, Beijing, China; 7 Key Laboratory of Epidemiology of Major Diseases (Peking University), Ministry of Education, Beijing, China

**Keywords:** major adverse cardiovascular events, real world study, regulatory science, target trial emulation, type 2 diabetes mellitus

## Abstract

**Introduction:**

Integrating real-world evidence (RWE) into regulatory decision-making requires validation against pivotal randomized controlled trials for the same estimand. We emulated the LEADER trial using Chinese claims data to evaluate liraglutide’s cardiovascular safety, assessing RWE-RCT concordance and examining the methodological adaptations and operational challenges encountered when emulating RCTs with claims data.

**Materials and methods:**

Using the Beijing Municipal Medical Insurance Database (2020–2024), we emulated the LEADER protocol. We identified new users of liraglutide and, as an active comparator, new users of DPP-4 inhibitors (DPP4i). The primary outcome was 3-point Major Adverse Cardiovascular Events. Propensity score matching was employed to balance baseline characteristics. Hazard Ratios were estimated using Cox proportional hazards models. Negative control outcomes (fractures and head injuries) and E-values were employed to assess the potential for unmeasured confounding.

**Results:**

The final cohort included 17, 772 patients (8, 886 per group). Over a median follow-up of 35 months, the incidence of MACE was 7.0% in the liraglutide group versus 7.8% in the DPP4i group. In the primary analysis, liraglutide was associated with a trend toward reduced cardiovascular risk (HR 0.90; 95% CI: 0.81, 1.01; *P* = 0.063), consistent with the effect size observed in the LEADER trial (HR 0.87), though not reaching statistical significance. However, sensitivity analysis adjusting for baseline comorbidities and drug exposure demonstrated a significant benefit (HR 0.90; 95% CI: 0.80, 1.01). The negative control analysis showed no association (HR 1.02; 95% CI: 0.93, 1.12), supporting the absence of unmeasured confounding.

**Conclusion:**

This target trial emulation study validated the cardiovascular safety profile of liraglutide observed in the LEADER trial. Liraglutide demonstrated non-inferiority to DPP-4 inhibitors for the risk of MACE. While the primary analysis for superiority showed a trend toward benefit, it did not reach statistical significance. However, sensitivity analyses suggested potential benefits and supporting the robustness of the safety findings.

## Introduction

Global regulatory initiatives increasingly seek to leverage real-world evidence (RWE) to support clinical and policy decision-making, while concerns about the internal validity of observational RWE studies remain, particularly regarding bias, data quality, and reproducibility, which can affect confidence in RWE findings (1, 2). Efforts to improve transparency, methodological rigor, and reproducibility—such as study registration, standardized protocols, and replication projects—are underway to enhance the reliability of RWE for regulatory and healthcare decisions (3–6). RWE, derived from real-world data (RWD) sources, provides evidence on drug safety, effectiveness, and utilization throughout the development and post-marketing phases. While the ICH E17 guideline established the global applicability of multiregional clinical trial data, the post-marketing phase still lacks robust, regulatory-grade evidence to support label expansions and safety surveillance (7). However, integrating RWE into regulatory decision-making is hindered by concerns regarding internal validity. To bridge this gap, observational studies based on local healthcare databases must demonstrate the capacity to produce causal inferences consistent with high-quality RCTs. This is best achieved through “replication” studies—using established RCT results as a benchmark to calibrate RWD sources and methodologies (5).

Liraglutide, a glucagon-like peptide-1 receptor agonist (GLP-1 RA), demonstrated cardiovascular benefits in the LEADER trial (NCT01179048), which serves as the pivotal study for its efficacy profile (8). In China, liraglutide has been used in clinical practice since 2011, generating extensive longitudinal data. Replicating the LEADER study using Chinese RWD offers an opportunity for methodological validation. Successful replication of the LEADER trial’s effect estimates would suggest that the underlying data source and analytical framework are sufficiently robust to support regulatory decisions when RCT evidence is unavailable.

Validating such a replication requires rigorous methodological frameworks to mitigate confounding and time-related biases. The target trial emulation (TTE) framework has emerged as a standard in causal inference, advocating that observational analyses emulate the protocol of a hypothetical randomized trial (9). This approach aligns with global initiatives like the FDA’s “RCT Duplicate” project (10). By applying this framework, researchers can systematically align eligibility criteria, treatment assignment, and follow-up procedures with those of the target trial.

In this study, we designed a target trial emulation based on the LEADER protocol utilized data from the Beijing Municipal Medical Insurance Database (BMMID). We selected DPP-4 inhibitors as an active comparator to minimize confounding by indication. The primary objective of this study is to evaluate the cardiovascular outcomes of liraglutide using the TTE framework based on Chinese claims data. Secondary to this, we aim to assess the concordance between RWE and RCT findings while critically examining the methodological and practical considerations on emulating RCTs investigating cardiovascular safety in type 2 diabetes mellitus (T2DM) patients.

## Materials and methods

### Study overview

We conducted a retrospective cohort study using a “Target Trial Emulation” framework to replicate the design of the LEADER trial (NCT01179048) within a real-world observational setting (11).Consistent with the principle of emulation, the LEADER trial’s protocol components were mapped to the administrative claims data (**Supplementary Table 1**). This study was reported in accordance with the TrAnsparent ReportinG of studies Emulating a Target trial (6). The completed TARGET checklist is provided in **Supplementary Table 2**. To enhance the transparency of this study, the protocol has been registered on the website of the International Society for Pharmacoeconomics and Outcomes Research (ISPOR, https://archive.org/details/osf-registrations-sgbu7-v1). In this retrospective observational study, we selected identical primary endpoints to those in the LEADER trial, aiming to align as closely as possible with its study population criteria, treatment strategies, and follow-up period. We employed propensity score matching and multivariate model correction as alternatives to randomization. Given the challenge of establishing a placebo group in observational research, we selected DPP-4 inhibitors (DPP4i) as the active comparator (10). This decision aligns with major clinical guidelines recognizing DPP-4 inhibitors as cardiovascularly neutral (12), thereby minimizing confounding by indication while providing a reference for assessing the relative efficacy of liraglutide.

### Data collection and drug exposure

All citizens with medical insurance are registered with a unique identification in China. The BMMID covers the medical data of thousands of hospitals and community clinics. The BMMID contains data on over 27 million insured individuals in the Beijing region, including structured data such as patient basic information, medical service details, prescriptions, and cost settlement. The database also includes outpatient visit information from nearly 8, 000 designated medical institutions in Beijing. Given its comprehensive scope, encompassing all medical treatment reimbursement activities for patients within the region, it ensures the continuity of patient care and the completeness of treatment information, thereby demonstrating excellent data coverage and quality. The medical insurance database uses standard medical terminology, with diagnoses coded ICD-10. This approach facilitates the effective identification of disease onset and comorbidities. Subsequent to the establishment of a data governance framework, a project database was generated, encompassing fundamental information, baseline information, treatment and efficacy information, and survival information. Data quality was ensured through multi-stage quality control, including encompassing the verification of the accuracy of the target population, comprehensive form verification, random patient dimension checks, and statistical analysis verification checks.

### Study population

The information used for grouping patient exposure is obtained from medical expense claim forms, ensuring accuracy. We included T2DM patients who had medical records in Beijing between January 1, 2020, and December 31, 2024. The exposure group consisted of T2DM who were prescribed liraglutide, whereas the reference group included patients prescribed DPP-4i during the same timeframe. The study size was determined by the availability of eligible patients in the database rather than a pre-specified power calculation. Our objective was to recruit a cohort size exceeding that of the original LEADER trial (n=9, 340) to ensure robust estimation. While we acknowledge that demonstrating superiority against an active comparator (DPP-4i) typically necessitates a different sample size than a placebo-controlled design, accurate *a priori* parameters (e.g., event rates and expected effect sizes for this specific comparison in the Chinese population) were unavailable to form a precise hypothesis. Consequently, we maximized the sample size by including the entire eligible population. *Post-hoc* power calculations were also avoided as they depend on the observed effect size and do not provide independent validation of the study design.We documented the concomitant use of other diabetes medications in both groups. In accordance with the LEADER Trial protocol and considering the feasibility of data variables, inclusion and exclusion criteria were established (see **Appendix Table S1**). New use of liraglutide or DPP-4i was defined as the first prescription of either drug class following a 6-month washout period without any prescriptions for liraglutide or DPP-4 inhibitors. For each patient, all payment dates for the target drugs were sorted chronologically. Starting from the first payment date for the study drugs, eligibility against the initial inclusion criteria was verified: if eligible, this payment date was designated as the index date (Day 1). If not eligible, the next payment date was checked for eligibility. As the study’s design was retrospective, no prior power calculation or sample size estimation was performed.

### Covariates, follow-up time, and outcomes

We included the following factors as potential covariates: age, gender, type of health insurance, education level, occupation, Charlson Comorbidity Index (CCI), comorbidities, history of baseline stroke, history of baseline myocardial infarction, prior medication history, duration of medication use, hospital grade at the baseline visit, number of baseline hospitalizations, number of baseline visits, and the interval from the initial diabetes diagnosis to the index date. In cases where multiple measurements for a variable existed prior to the index date, the measurement closest to the index date was selected as the baseline value. The observation period was defined as commencing on the day following the index date and continuing until the last recorded date in the dataset. The primary outcome is a composite endpoint referred to as 3-point major adverse cardiovascular events (3P-MACE), which is defined as the first occurrence of cardiovascular disease-related death, myocardial infarction (MI), and stroke, as determined by ICD coding. Secondary outcomes include individual events of cardiovascular disease-related death, MI, and stroke.

### Statistical analyses

We reported descriptive statistics for baseline covariates. Mean and SD were provided for continuous variables, and frequency and percentage were reported for categorical variables. To mitigate potential confounding and address imbalances in baseline covariates between the study groups, we performed propensity score matching (PSM). A logistic regression model was implemented to calculate propensity scores, with stepwise selection utilized to identify the optimal set of matching variables and enhance comparability between groups. Patients were subsequently matched 1:1 via the nearest neighbor method without replacement, applying a stringent caliper of 0.05. The adequacy of covariate balance between liraglutide and DPP-4i users was evaluated using the standardized mean difference (SMD), where an SMD < 0.1 was pre-specified as the threshold for achieving a comparable balance in baseline characteristics.

The treatment effect was estimated using Cox proportional hazards models to calculate Hazard Ratios (HR) and 95% confidence intervals (CI). The Cox proportional hazards model incorporated variables recommended by medical experts, including age, gender, ethnicity, insurance type, and medical history as adjustment factors in the primary analysis. Kaplan-Meier curves were generated to visualize the cumulative incidence of events over time. To fully emulate the LEADER statistical analysis plan (8), we adopted an identical sequential hypothesis testing strategy:

1. **Non-inferiority Test:** Confirming if the upper bound of the 95% CI for the HR was < 1.3. This margin was pre-specified in accordance with the US FDA’s 2008 Guidance for Industry on evaluating cardiovascular risk in new antidiabetic therapies and the EMA’s corresponding guidelines, which established 1.3 as the regulatory standard for excluding unacceptable cardiovascular risk (13).
2. **Superiority Test:** If non-inferiority was established, testing if the upper bound of the 95% CI was < 1.0.

### Sensitivity analyses

To ensure the robustness of our primary findings and to address potential sources of bias, we implemented a comprehensive sensitivity analysis framework structured across four methodological dimensions as detailed in **Supplementary Table 3**. To mitigate inherent data source biases, we conducted a subgroup analysis of verified medication users with documented glycemic testing (e.g., HbA1c) prior to the index prescription and refined the primary outcome by reclassifying all-cause death within 30 days of a cardiovascular event as cardiovascular-related death. Treatment adherence was further scrutinized through a per-protocol analysis that censored patients upon medication discontinuation and restricted the cohort to individuals with over 180 days of continuous use. To evaluate the consistency of treatment effects and account for clinical heterogeneity, we performed stratified analyses based on age, sex, and baseline cardiovascular history, while also validating results within a primary prevention cohort. Statistical modeling assumptions were tested by employing Inverse Probability of Treatment Weighting (IPTW) with trimming of extreme weights (<1% or >99%) and by systematically modifying covariate selection to assess model dependency. Finally, to account for unmeasured confounding, we utilized negative control outcomes—such as fractures and head injuries—as falsification tests and calculated E-values to quantify the minimum strength of association required for an unobserved confounder to nullify the observed treatment effect.

All statistical analyses were performed using R 4.3.1.

## Results

### Study population

**Figure 1:**
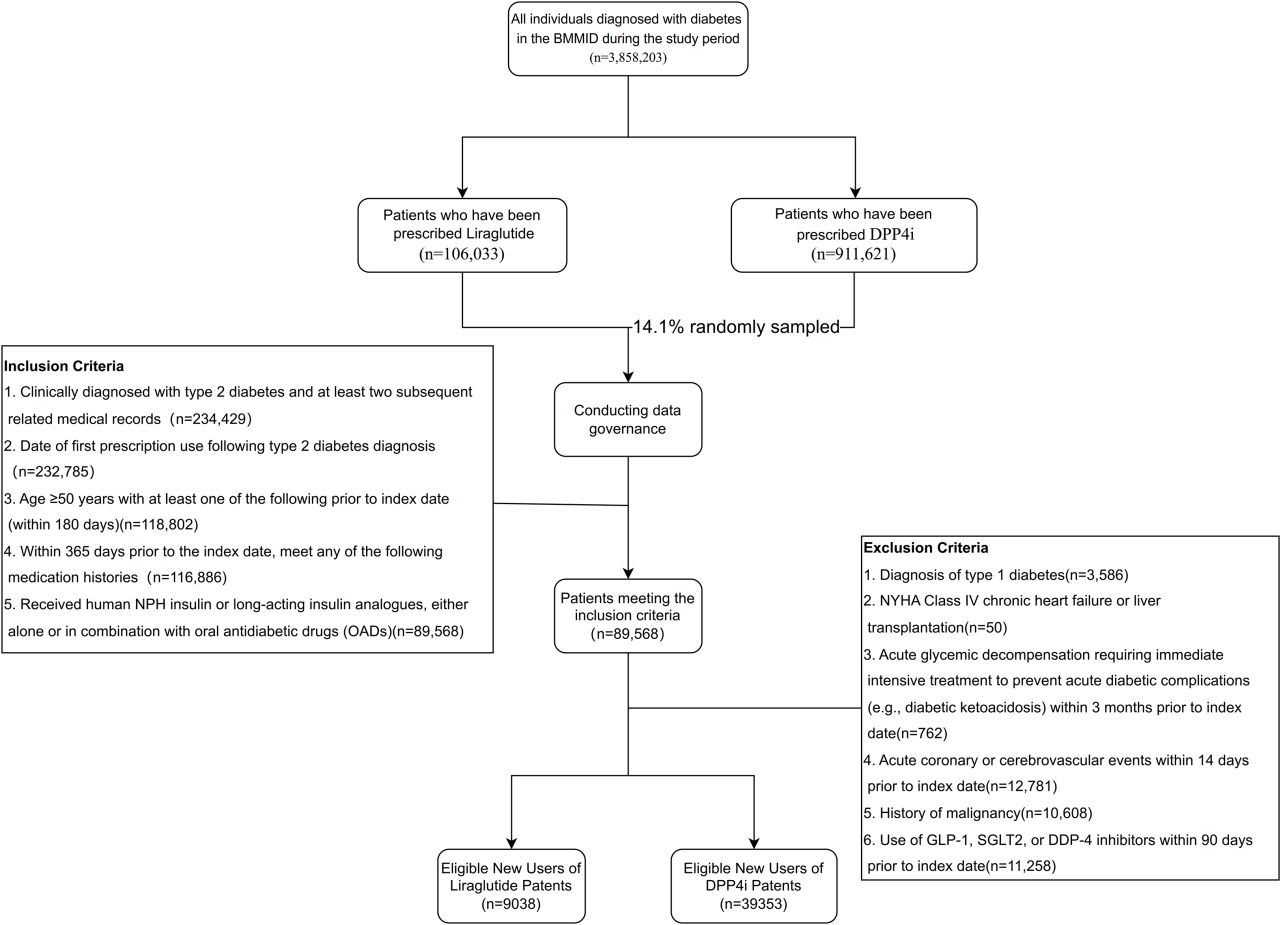
Flow diagram for study participants..

The BMMID encompasses a total of 3, 858, 203 patients diagnosed with diabetes. Within this database, the diabetes specialty segment includes 106, 033 patients who have received prescriptions for liraglutide and 911, 621 patients prescribed DPP-4 inhibitors. From a feasibility standpoint, data governance was conducted on a randomly selected cohort of 128, 000 patients receiving DPP-4 inhibitor prescriptions, who were subsequently matched to the liraglutide cohort. Based on established inclusion and exclusion criteria, 48, 391 patients satisfied the protocol requirements (see **Figure 1**). Prior to propensity score (PS) matching, the standardized mean difference (SMD) values revealed imbalances between the two groups across various dimensions, including demographic characteristics, clinical features, prior treatment history, and first-time medication use. After matching for variables such as age, insurance type, comorbidities, prior treatment history, and duration of medication use, all observed variables were effectively controlled. Ultimately, the combined patient cohort consisted of 17, 772 subjects, with 8, 886 patients in each group. Demographics and baseline characteristics.

**Supplementary Table 4** summarizes the demographic characteristics, clinical comorbidities, and medication histories of the study participants before and after PS matching. Prior to matching, substantial imbalances were observed between the liraglutide (N = 9, 038) and DPP-4 inhibitor (N = 39, 353) cohorts across several key variables, notably in age (SMD = 0.285), health insurance type (SMD = 0.587), and previous insulin use (SMD = 0.416)2. The PS matching process successfully addressed these initial discrepancies, as evidenced by the reduction of nearly all SMDs to below the 0.1 threshold, indicating a well-balanced distribution of covariates. For instance, post-matching SMDs for age, gender, Charlson Comorbidity Index (CCI), and history of cardiovascular or renal diseases were all recorded at ≤ 0.021.

### MACE outcome

**Figure 2:**
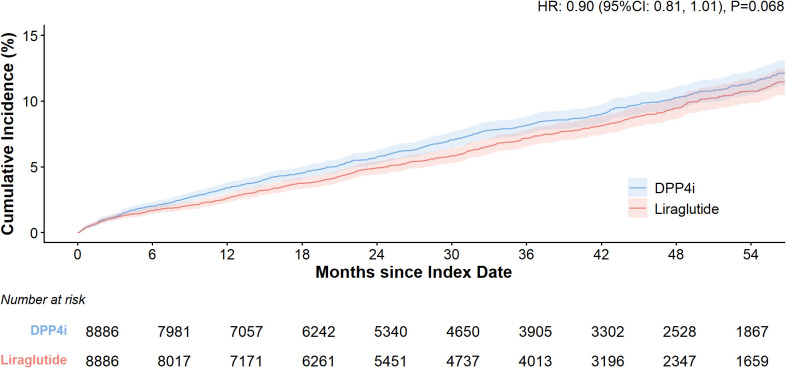
Kaplan-Meier curves for the cumulative incidence of MACE.

During the observation period, 619 patients in the liraglutide group experienced 3-point MACE (7.0%), while 690 patients in the DPP-4 inhibitor group experienced 3-point MACE (7.8%). The median time to the first event was slightly longer in the liraglutide group compared to the DPP-4 inhibitor group (35.3 months vs. 34.4 months). The two-year event-free rates were 95.1% in the liraglutide group and 94.3% in the DPP-4 inhibitor group. After adjusting for potential confounders—including age, education level, insurance type, occupation, Charlson Comorbidity Index (CCI), hospital grade at visit, number of hospitalizations, number of visits, baseline stroke occurrence, time interval from initial diabetes diagnosis to index date, index year, comorbidities, and prior treatment history—liraglutide was associated with a 10% reduction in cardiovascular event risk compared to DPP-4 inhibitors; however, this difference did not reach statistical significance (HR: 0.90; 95% CI 0.81, 1.01) (**Table 1**, **Figure 2**).

**Table 1:** Primary analysis of MACE events after PSM matching.

Events	Liraglutide	DPP4i
Primary analysis		
N	8886	8886
major adverse cardiovascular event (MACE)	619 (7.0)	690 (7.8)
Non-fatal stroke	341 (3.8)	367 (4.1)
Non-fatal myocardial infarction	235 (2.6)	283 (3.2)
Cardiovascular-related death	98 (1.1)	109 (1.2)
Hazard ratio (HR)	0.90	
HR 95%CI	0.81, 1.01	
P value	0.063	
2-year event-free survival rate (95% CI)	95.1 (94.6, 95.6)	94.3 (93.7, 94.8)
3-year event-free survival rate (95% CI)	92.8 (92.2, 93.5)	91.9 (91.2, 92.5)
4-year event-free survival rate (95% CI)	90.6 (89.8, 91.4)	89.7 (88.9, 90.6)
Median event-free survival time (95% CI) (months)	35.3 (34.6, 35.8)	34.4 (33.6, 35.3)

### Sensitivity analyses and data robustness

**Figure 3:**
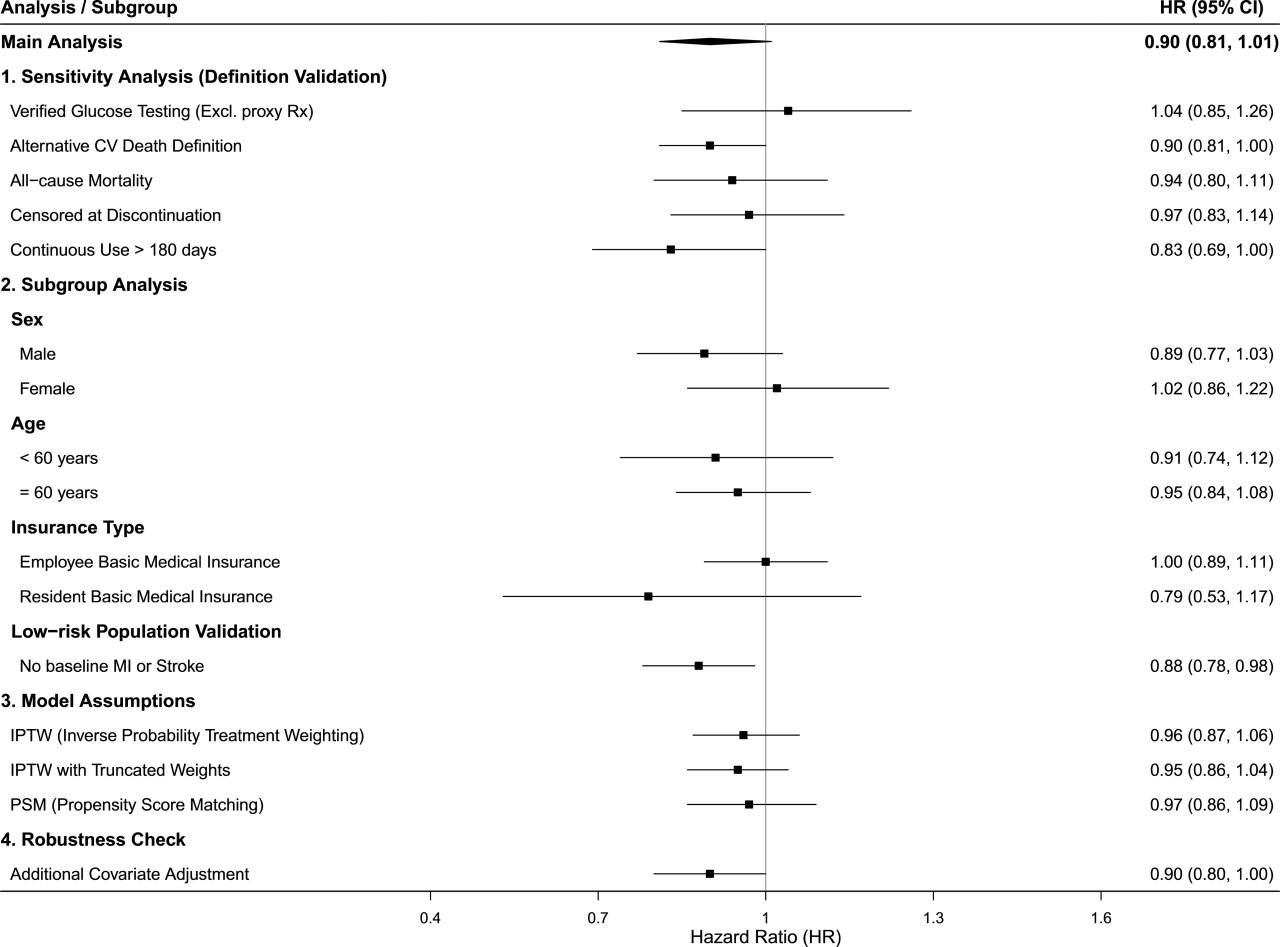
Forest plot of sensitivity analysis.

The results of the pre-specified sensitivity and subgroup analyses, summarized in the forest plot (**Figure 3**), demonstrate the robustness of the primary findings across various clinical and methodological scenarios. The hazard ratio for the main analysis (0.90, 95% CI: 0.81, 1.01) remained remarkably stable when tested against alternative modeling assumptions, including Inverse Probability of Treatment Weighting (HR 0.96, 95% CI: 0.87, 1.06) and additional covariate adjustments (HR 0.90, 95% CI: 0.80, 1.01). Subgroup stratifications by age and sex further confirmed the consistency of the treatment effect, showing no significant deviation from the primary estimate. Notably, a more pronounced trend toward cardiovascular benefit was observed in the “continuous use > 180 days” cohort (HR 0.83, 95% CI: 0.69, 1.01) and within the low-risk population validation (HR 0.88, 95% CI: 0.78, 0.98), where the association reached statistical significance.

### Assessment of unmeasured confounding

Fracture and head injury were utilized as negative control outcomes to assess potential unmeasured confounding. No significant differences were observed between the liraglutide and DPP-4i groups for either fracture (HR 1.02; 95% CI 0.93, 1.12; *P* = 0.619) or head injury (HR 1.00; 95% CI 0.92, 1.09; *P* = 0.936). Furthermore, quantitative bias analysis yielded an E-value of 1.9 based on the non-inferiority margin of 1.3 and the primary MACE estimate (**Table 2**). This implies that an unmeasured confounder (e.g., smoking status or BMI) would need to be associated with both treatment assignment and the outcome by a risk ratio of at least 1.90 to nullify the conclusion of cardiovascular safety (non-inferiority).

**Table 2:** PSM-matched negative control outcome estimates.

Event	Liraglutide (N = 8886)	DPP4i (N = 8886)
Fracture	915 (10.3)	893 (10.1)
Hazard ratio (HR)	1.0	
HR 95%CI	(0.9, 1.1)	
*P* value	0.6	
2-year event-free survival rate (95% CI)	92.9 (92.3, 93.5)	93.0 (92.4, 93.6)
3-year event-free survival rate (95% CI)	89.4 (88.6, 90.1)	90.2 (89.5, 90.9)
4-year event-free survival rate (95% CI)	86.5 (85.6, 87.4)	87.1 (86.2, 88.0)
Median event-free survival time (95% CI) (months)	35.3 (34.6, 35.9)	34.3 (33.5, 35.1)
Head injuries	1046 (11.8)	1071 (12.1)
Hazard ratio (HR)	1.0	
HR 95%CI	(0.9, 1.1)	
*P* value	0.9	
2-year event-free survival rate (95% CI)	91.6 (91.0, 92.2)	91.9 (91.3, 92.5)
3-year event-free survival rate (95% CI)	88.9 (88.2, 89.7)	89.3 (88.5, 90.0)
4-year event-free survival rate (95% CI)	85.6 (84.7, 86.5)	86.1 (85.2, 87.0)
Median event-free survival time (95% CI) (months)	36.2 (35.6, 41.3)	35.5 (34.8, 36.3)

### Comparison with LEADER trial

**Table 3** shows that compared with the LEADER Trial, the liraglutide group in this study included more patients, a higher proportion of women (46.6% vs 35.5%), and a shorter duration of diabetes (2.3 years vs 12.8 years). The primary analysis results of this study showed a similar risk ratio for MACE outcomes compared to the LEADER Trial (0.90 [95% CI: 0.81, 1.01] vs 0.87 [95% CI: 0.78, 0.97]), with a lower event rate in the liraglutide group than in the LEADER Trial (6.97% vs 13%). Among the three cardiovascular endpoints, cardiovascular-related deaths constituted a relatively smaller proportion in this study (98/619 vs. 219/608).

**Table 3:** Demographic characteristics and primary MACE results: LEADER vs. our study.

Variable	LEADER (FAS)		Our study	
	Liraglutide	Placebo	Liraglutide	DPP4i
	N=4668	N=4672	N=8886	N=8886
Age (years)				
Mean (SD)	64.2 (7.2)	64.4 (7.2)	63.8 (8.6)	63.9 (8.5)
Age (years)				
(18, 64)	2512 (53.8)	2499 (53.5)	5047 (56.8)	5109 (57.5)
(65, 84)	2139 (45.8)	2148 (46.0)	3661 (41.2)	3595 (40.5)
>=85	17 (0.4)	25(0.5)	178 (2.0)	182 (2.0)
Sex, n (%)				
Male	3011 (64.5)	2992 (64.0)	4746 (53.4)	4790 (53.9)
Female	1657 (35.5)	1680 (36.0)	4140 (46.6)	4096 (46.1)
Duration of diabetes (years)				
Mean (SD)	12.8 (8.0)	12.9 (8.1)	2.3 (1.8)	2.3 (1.8)
MACE primary analysis				
MACE	608 (13.0)	694 (14.9)	619 (7.0)	690 (7.8)
HR (95%CI)	0.87 (0.78, 0.97)		0.90 (0.81, 1.01)	
Non-fatal stroke	159 (3.4)	177 (3.8)	341 (3.8)	367 (4.1)
HR (95%CI)	0.89 (0.75, 1.03)		0.92 (0.79, 1.07)	
Non-fatal myocardial infarction	281 (6.0)	317 (6.8)	235 (2.6)	283 (3.2)
HR (95%CI)	0.88 (0.75, 1.03)		0.85 (0.71, 1.01)	
Cardiovascular death	219 (4.7)	278 (6.0)	98 (1.1)	109 (1.2)
HR (95%CI)	0.78 (0.66, 0.93)		0.97 (0.74, 1.29)	

## Discussion

This study provides a robust real-world validation of liraglutide’s cardiovascular safety in a large Chinese cohort, utilizing a Target Trial Emulation (TTE) framework to minimize inherent observational biases. Our primary analysis demonstrated that liraglutide was associated with a non-inferior risk of 3-point MACE compared to DPP-4 inhibitors (HR 0.90, 95% CI 0.81, 1.01). Although the risk reduction did not reach statistical superiority (*P* = 0.063), the direction and magnitude of the effect estimate (HR 0.90) are highly concordant with the landmark LEADER trial (HR 0.87). We explicitly clarify that while this data is sufficient for confirming non-inferiority (safety), it requires cautious interpretation for superiority (efficacy) claims due to the missing clinical nuances (e.g., lab data) and outcome ascertainment limitations. These findings also suggest that when rigorous emulation frameworks are applied, administrative claims data can yield effect estimates consistent with randomized controlled trials (RCTs), thereby supporting the integration of real-world evidence (RWE) into regulatory decision-making.

While our study successfully emulated the key protocol components of the LEADER trial (as detailed in **Supplementary Table 1**), a nuanced interpretation of the findings requires distinguishing between the target estimand of the RCT and the emulated estimand feasible within observational data. We identified specific deviations across four estimand attributes, primarily driven by the nature of real-world data (RWD) and Chinese clinical practice:

Primarily, regarding the treatment attribute, the most fundamental deviation is the choice of comparator. Unlike LEADER trial compared liraglutide to a placebo (added to standard of care), our study utilized DPP-4 inhibitors as an active comparator. This shift transforms the estimand from assessing “efficacy against standard of care alone” to “comparative effectiveness against a cardiovascular-neutral active agent.” Notably, emerging evidence suggests that DPP-4i may possess pleiotropic protective effects, potentially mediated through improved endothelial function, reduction of oxidative stress, and anti-inflammatory pathways (14–16). Several meta-analyses have indicated that DPP-4i might slightly reduce the incidence of MACE compared to sulfonylureas, even if the benefit remains non-significant compared to placebo (15, 17). Consequently, by evaluating liraglutide against DPP-4i rather than a placebo, our study adopted a more conservative benchmark. This higher comparative baseline likely accounts for the lack of statistical superiority, as detecting incremental benefits over an already cardiovascular-safe therapy is inherently more challenging.

Secondarily, in terms of the variable attribute (endpoint ascertainment), deviations arose from data granularity. While LEADER employed a rigorous adjudication committee to capture all cardiovascular events, the BMMID database primarily captures in-hospital mortality. This under-ascertainment of out-of-hospital cardiovascular deaths represents a deviation in the variable definition, explaining the 3-point MACE rate in our study (7.0%) compared to that reported in LEADER (13.0%). Given that cardiovascular death was a key driver of significance in the original trial (8), this “granularity gap” inherently constrained our statistical power to reach the same significance threshold, even though the point estimate (HR) remained consistent.

Thirdly, concerning intercurrent events, we emulated the Intention-to-Treat (ITT) effect (treatment policy strategy) in the primary analysis. However, a significant implementation deviation exists due to real-world adherence patterns. Unlike the protocol-driven environment of an RCT, real-world clinical practice often involves frequent treatment discontinuation, which inevitably dilutes the observed biological efficacy. This is corroborated by our per-protocol sensitivity analysis, where restricting the analysis to patients with continuous use (>180 days) revealed a significant protective effect (HR 0.83), closely matching the LEADER efficacy signal. This discrepancy underscores that the cardiovascular benefits of liraglutide are highly adherence-dependent, providing vital guidance for clinicians to prioritize treatment persistence in the Chinese population (18, 19).

Lastly, regarding the population attribute, the real-world cohort in our study represented a more advanced disease profile. Despite propensity score matching, the higher baseline insulin usage in the liraglutide group (63.2% vs. 42.8%) suggests that in Chinese clinical practice, liraglutide is often reserved for patients with a longer history of glycemic failure or those who have failed multiple oral agents. Additionally, the initiation of GLP-1 RAs occurs significantly later in the disease trajectory compared to the early intervention model seen in RCTs (20, 21). These disparities highlight the inherent challenges in replicating RCT results within the complexities of routine clinical care, where treatment inertia and patient heterogeneity are prevalent.

Beyond the core emulation, our analysis of the primary prevention cohort further enhances the generalizability of the original trial’s findings within the Chinese context. While the original LEADER trial focused on a high-risk population with established cardiovascular or renal disease, our sensitivity analysis demonstrated significant cardiovascular protection (HR 0.88, 95% CI 0.78, 0.98) in a broader, lower-risk population within the BMMID. This suggests that liraglutide may offer protective benefits earlier in the disease progression for Chinese patients. This validation across different risk strata underscores the potential of liraglutide as a versatile tool for early cardiovascular intervention, suggesting that the “cardioprotective” threshold for GLP-1 RAs might be lower in certain ethnic populations or clinical contexts than previously assumed (22).

The reliability of these clinical insights is further underpinned by multiple safeguards. Quantitative bias analysis yielded an E-value of 1.90 for the non-inferiority margin, indicating that to overturn the current conclusion, an unmeasured confounder would require a risk ratio of at least 1.90 associated with both the treatment assignment and the outcome. Considering the lack of association observed in our negative control outcomes (fractures and head injuries), the probability that such a substantial and systematically imbalanced confounder exists within the matched cohort to nullify the observed effects is objectively minimal.

We acknowledge the limitations inherent in administrative data, such as the lack of granular laboratory values (e.g., HbA1c, lipid profiles), could not fully capture out-of-hospital cardiovascular events, particularly deaths and the potential for unobserved bias. However, the successful balancing of all observed covariates via propensity score matching and the consistent results across multiple sensitivity analyses suggest that the comparative treatment effects remain reliable. These results provide robust evidence supporting the cardiovascular safety of liraglutide in the Chinese population while demonstrating the feasibility of using TTE to generate high-quality RWE from administrative databases.

## Conclusion

In conclusion, this study not only provides real-world evidence supporting the cardiovascular safety (non-inferiority) of liraglutide in Chinese patients but also demonstrates the capacity of the target trial emulation framework to bridge the gap between observational studies and randomized trials. While deviations in the comparator and data granularity warrant cautious interpretation of superiority, and lack direct baseline comparisons with the LEADER trial due to the common variables, the findings reinforce the potential of high-quality RWE to complement traditional trials. The rigorous internal matching and consistent sensitivity analyses validate our findings. By successfully emulating the effect estimates of a global RCT, this study reinforces the potential of high-quality RWE to complement traditional trials in drug lifecycle management and regulatory decision-making. Future research should leverage broader datasets with integrated laboratory results to further substantiate these benefits across diverse populations.

## Data Availability

The data analyzed in this study is subject to the following licenses/restrictions: The datasets presented in this article are not readily available because of the policy request. Requests to access the datasets should be directed to the corresponding author. Requests to access these datasets should be directed to zhaojun@cde.org.cn
