# Supplementary material for: Emulating the LEADER trial in China: a regulatory science case study on non-interventional research
Source: Front Endocrinol (Lausanne). 2026 Mar 12;17:1777954. doi: 10.3389/fendo.2026.1777954 (PMC13017370; doi:10.3389/fendo.2026.1777954)
Supplement: Supplementary file 1 [file DataSheet1.pdf]

Supplementary Material for Emulating the LEADER Trial in China A Regulatory Science Case Study on Non-interventional Research

Contents

Item	Description	Page
Supplementary Table S1	Key Elements of the Leader Trial and Emulation Study Protocol	2-5
Supplementary Table S2	The TARGET Guideline Checklist.	4-9
Supplementary Table S3	Bias Identification and Sensitivity Analysis Strategy Matrix	10-11
Supplementary Table S4	Demographic Characteristics, Concomitant Medications, and Medical History of Study Populations Before and After PS Matching	12-18

Supplementary Table S1: Key Elements of the Leader Trial and Emulation Study Protocol

Key Elements	Leader Trial	Emulation Study
Eligibility Criteria	**Inclusion Criteria**<br>1. Informed consent obtained before any trial-related activities. (Trial-related activities are any procedure that would not have been performed during normal management of the subject)<br>2. Men or women with type 2 diabetes<br>3. Age ≥ 50 years at screening and at least one of the below criteria (from **a** to **h** below):<br>a) prior myocardial infarction<br>b) prior stroke or prior transient ischaemic attack (TIA)<br>c) prior coronary, carotid or peripheral arterial revascularisation<br>d) >50% stenosis on angiography or other imaging of coronary, carotid or lower extremity arteries<br>e) history of symptomatic coronary heart disease documented by positive exercise stress test<br>or any cardiac imaging, or unstable angina with ECG changes<br>f) asymptomatic cardiac ischemia documented by positive nuclear imaging test or exercise<br>test or dobutamine stress echo<br>g) chronic heart failure NYHA class II-III<br>h) chronic renal failure, defined as glomerular filtration rate < 60 mL/min/1.73 m^2 per Modification of Diet in Renal Disease (MDRD) or < 60 mL/min per Cockroft-Gault formula<br><br>OR	**Inclusion Criteria**<br>1. Clinically diagnosed with type 2 diabetes and at least two subsequent related medical records<br>2. Date of first prescription use following type 2 diabetes diagnosis<br>3. Age ≥50 years with at least one of the following prior to index date (within 180 days):<br>i. Comorbidities of heart disease, cerebrovascular disease, or kidney disease<br>ii. MACE events (non-fatal myocardial infarction, non-fatal stroke)<br>4. Within 365 days prior to the index date, meet any of the following medication histories:<br>i. No use of antidiabetic drugs<br>ii. Received treatment with one or more oral antidiabetic drugs (OADs)<br>5. Received human NPH insulin or long-acting insulin analogues, either alone or in combination with oral antidiabetic drugs (OADs)<br>**Exclusion Criteria**<br>1. Diagnosis of type 1 diabetes<br>2. NYHA Class IV chronic heart failure or liver transplantation within 180 days prior to index date<br>3. Acute glycemic decompensation requiring immediate intensive treatment to prevent acute diabetic complications (e.g., diabetic ketoacidosis) within 3 months prior to index date<br>4. Acute coronary or cerebrovascular events within 14 days prior to index date<br>5. History of malignancy

Key Elements	Leader Trial	Emulation Study
	Age ≥ 60 years at screening and meeting at least one of the below criteria (from **a** to **d** below)<br>a) microalbuminuria or proteinuria<br>b) hypertension and left ventricular hypertrophy by ECG or imaging<br>c) left ventricular systolic or diastolic dysfunction by imaging<br>d) ankle/brachial index < 0.9<br>4. Anti-diabetic drug naïve or treated with one or more oral anti-diabetic drugs or treated with human NPH insulin or long-acting insulin analogue, alone or in combination with OAD(s)<br>5. $HbA_{1c} \geq 7.0\%$ at screening<br>**Exclusion Criteria**<br>1. Type 1 diabetes<br>2. Use of a GLP-1 receptor agonist (exenatide, liraglutide or other) or pramlintide or any DPP-4 inhibitor within the 3 months prior to screening<br>3. Use of insulin other than human NPH insulin or long-acting insulin analogue within 3 months prior to screening. Short-term use of other insulin during this period in connection with intercurrent illness is allowed at the investigator's discretion.<br>4. Acute decompensation of glycaemic control requiring immediate intensification of treatment to prevent acute complications of diabetes (e.g., diabetes ketoacidosis) in the previous 3 months<br>5. An acute coronary or cerebrovascular event in the previous 14 days<br>6. Currently planned coronary, carotid or peripheral artery revascularisation	6. Use of GLP-1, SGLT2, or DDP-4 inhibitors within 90 days prior to index date.

Key Elements	Leader Trial	Emulation Study
	7. Chronic heart failure NYHA class IV<br>8. Current continuous renal replacement therapy<br>9. Estimated glomerular filtration rate (eGFR) (as per MDRD) < 30 mL/min/1.73m^2 at screening. The criterion is applicable after a target number of 220 subjects with eGFR < 30 mL/min are randomised<br>10. End-stage liver disease, defined as the presence of acute or chronic liver disease and recent history of one or more of the following: ascites, encephalopathy, variceal bleeding, bilirubin ≥ 2.0 mg/dL, albumin level ≤ 3.5 g/dL, prothrombin time ≥ 4 seconds prolonged, international normalised ratio (INR) ≥1.7 or prior liver transplant<br>11. A prior solid organ transplant or awaiting solid organ transplant<br>12. Malignant neoplasm requiring chemotherapy, surgery, radiation or palliative therapy in the previous 5 years. Patients with intraepithelial squamos cell carcinoma of the skin (Bowen's disease) treated with topical 5FU and subjects with basal cell skin cancer are allowed to enter the trial<br>13. Family or personal history of multiple endocrine neoplasia type 2 (MEN2) or familial medullary thyroid carcinoma (FMTC)<br>14. Personal history of non-familial medullary thyroid carcinoma<br>15. Any acute condition or exacerbation of chronic condition that would in the Investigator's opinion interfere with the initial trial visit schedule and procedures	

Key Elements	Leader Trial	Emulation Study
	16. Known or suspected hypersensitivity to trial product(s) or related products<br>17. Known use of non prescribed narcotics or illicit drugs<br>18. Simultaneous participation in any other clinical trial of an investigational agent. Participation in a clinical trial with investigational stent(s) is allowed<br>19. Previous participation in this trial. Participation is defined as randomised<br>20. Females of childbearing potential who are pregnant, breast-feeding or intend to become pregnant or are not using adequate contraceptive methods (adequate contraceptive measures as required by local law or practice)<br>21. Receipt of any investigational medicinal product (IMP) within 30 days prior to this trial.	
Treatment Strategy	Liraglutide vs placebo,both administered in addition to the subject's standard treatment.	Liraglutide vs DPP4i, both under usual medical care.
Treatment Allocation	Patients were randomly assigned in a 1:1 ratio to receive either liraglutide or placebo.	Patients were grouped based on the glucose-lowering medication used at the time of initial eligibility and matched 1:1 using propensity score matching.
Follow-up	Randomize until patients experience a 3pMACE, or censor patients without a 3pMACE based on their last recorded known survival date	From medication administration to the occurrence of MACE events in patients, or by censoring patients without 3pMACE based on their final visit records.
Outcome	3p MACE,Time from randomisation to first occurrence of cardiovascular death, non-fatal myocardial infarction, or non-fatal stroke (a composite cardiovascular outcome). The percentage of subjects experiencing a first event of cardiovascular death, non-fatal myocardial infarction, or non-fatal stroke (a composite cardiovascular outcome) is presented.	Same as LEADER
Causal Comparison	Intention-to-treat analysis under random assignment	Counterfactual Frameworks in Observational Studies

Note: While the design elements were mapped as closely as possible, necessary deviations regarding the comparator (Active vs. Placebo) and outcome ascertainment (In-hospital vs. Adjudicated) are discussed in detail in the Discussion section of the main text.

Supplementary Table S2: The TARGET Guideline Checklist

<table>
<tr><th>Item no.</th><th></th><th colspan="3">Checklist item</th><th colspan="2">Location reported</th></tr>
<tr><td colspan="7">Abstract</td></tr>
<tr><td>1</td><td>a</td><td colspan="3">Identify that the study attempts to emulate a target trial using observational data. State the study objectives and briefly summarize the specified target trial.</td><td colspan="2">Title; Abstract - Methods</td></tr>
<tr><td></td><td>b</td><td colspan="3">Report the data sources used for emulation.</td><td colspan="2">Abstract - Materials and Methods; Abstract - Conclusion</td></tr>
<tr><td></td><td>c</td><td colspan="3">Summarize key assumptions, statistical methods, findings and conclusions.</td><td colspan="2">Abstract - Methods, Results, Conclusion</td></tr>
<tr><td colspan="7">Introduction</td></tr>
<tr><td>2</td><td>Background</td><td colspan="3">Describe the scientific background of the study and the gap in knowledge.</td><td colspan="2">Introduction (Paragraphs 1-3)</td></tr>
<tr><td>3</td><td>Causal question</td><td colspan="3">Summarize the causal question.</td><td colspan="2">Introduction (Last paragraph)</td></tr>
<tr><td>4</td><td>Rationale</td><td colspan="3">Describe the rationale for emulating a target trial with the available data. Cite randomized trials informing the design of the target trial if applicable.</td><td colspan="2">Introduction (Paragraphs 4-6)</td></tr>
<tr><td colspan="7">Methods</td></tr>
<tr><td>5</td><td>Data sources</td><td colspan="3">Cite the data sources contributing to the analyses and for each one describe the following: original purpose, type, the geographic locations, setting and time-period. If relevant, describe how the data were linked or pooled.</td><td colspan="2"></td></tr>
<tr><td>6</td><td colspan="2">Target trial specification
Specify the components of the target trial protocol that would answer the causal question.</td><td>7</td><td>Target trial emulation
Describe how the components of the target trial protocol were emulated with the observational data, including how all variables were measured or ascertained.</td><td>Location item 6 (specification) reported</td><td>Location item 7 (emulation) reported</td></tr>
<tr><td></td><td colspan="2">Eligibility criteria</td><td colspan="2">Eligibility criteria</td><td rowspan="2">Supplementary Table S1</td><td rowspan="2">Supplementary Table S1</td></tr>
<tr><td></td><td>a</td><td>Describe the eligibility criteria.</td><td>a</td><td>Describe how the eligibility criteria were operationalized with the data.</td></tr>
</table>

**Treatment strategies**		**Treatment strategies**		Supplementary Table S1	Supplementary Table S1
b	Describe the treatment strategies that would be compared.	b	Describe how the treatment strategies were operationalized with the data.		
**Assignment procedures**		**Assignment procedures**		Supplementary Table S1	Supplementary Table S1
c	Report that eligible individuals would be randomly assigned to treatment strategies and may be aware of their treatment allocation.	c	Describe how assignment to treatment strategies was operationalized with the data.		
**Follow-up**		**Follow-up**		Supplementary Table S1	Supplementary Table S1
d	Clarify that follow-up would start at time of assignment to the treatment strategies. Specify when follow-up would end.	d	Clarify that follow-up starts at the time individuals were assigned to the treatment strategies. Describe how the end of follow-up was operationalized with the data.		
**Outcomes**		**Outcomes**		Supplementary Table S1	Supplementary Table S1
e	Describe the outcomes.	e	Describe how the outcomes were operationalized with the data.		
**Causal contrasts**		**Causal contrasts**		Supplementary Table S1	Supplementary Table S1
f	Describe the causal contrasts of interest, including effect measures.	f	Describe how the causal contrasts were operationalized with the data, including effect measures.		
**Identifying assumptions**		**Identifying assumptions**		Materials and methods - Statistical Analyses (HR, Non-inferiority/Superiority tests)	Materials and methods - Statistical Analyses (HR, Non-inferiority/Superiority tests)
g	Describe assumptions that would be made to identify each causal estimand. Describe the variables, if any, related to these assumptions.	g.i	For each causal estimand, describe assumptions made to identify it, including assumptions regarding baseline confounding due to lack of randomization.		
		g.ii	Describe how the variables related to these assumptions were operationalized with the data		Materials and methods - Study overview (Active comparator assumption); Materials and methods - Sensitivity Analyses (Unmeasured

				confounding/Negative controls)
	**Data analysis plan**	**Data analysis plan**		
	h For each causal estimand, describe the data analysis procedures and any associated statistical modelling assumptions, including approaches for handling missing data.	h.i For each causal estimand, describe the data analysis procedures and any associated statistical modelling assumptions, including approaches for handling missing data.		Materials and methods - Statistical Analyses
		h.ii For each causal estimand, describe any additional analyses conducted to assess the sensitivity of the results to the choice of operationalizations, assumptions and analysis.		Materials and methods - Statistical Analyses
**Results**				
**8**	Participant selection	Report numbers of individuals assessed for eligibility, eligible, and assigned to each treatment strategy. A flow diagram is strongly recommended.		Results - Study Population; Figure 1
**9**	Baseline data	Describe the distribution of characteristics of individuals at baseline, by treatment strategy.		Results - Demographics and baseline characteristics; Appendix table S4
**10**	Follow-up	Summarize length of follow-up and describe reasons for end of follow-up for each treatment strategy and causal contrast.		Results - MACE outcome (Median follow-up)
**11**	Missing data	Describe the frequency of missing data in all variables, by treatment strategy when applicable.		Materials and methods - Data Collection and Drug Exposure (Data quality/governance); Results - Study Population (Matching results)
**12**	Outcomes	Describe the frequency or distribution of each outcome, by treatment strategy.		Results - MACE outcome; Table 2
**13**	Effect estimates	Report the effect estimates for each causal contrast with corresponding measures of precision, including both absolute and relative measures of effect, when applicable.		Results - MACE outcome; Table 2
**14**	Additional analyses	Report results of all analyses to assess the sensitivity of the estimates to choices in operationalizations, assumptions and analysis.		Results - Sensitivity Analyses and Data Robustness; Figure 3
**Discussion**				
**15**	Interpretation	Provide an interpretation of the key findings.		Discussion - Paragraph 1,3

**16**	Limitations	Discuss the limitations of the study considering differences between the target trial and its emulation and the plausibility of assumptions, including assumptions regarding baseline confounding due to lack of randomization.	Discussion - Paragraph 2
**Other information**			
**17**	Ethics	Provide the institutional research board or ethics committee that approved the study and approval numbers, if relevant.	Ethics statement
**18**	Registration	State whether, when and where the study protocol was registered.	Materials and methods - Study overview (ISPOR registration)
**19**	Sharing of study materials	Provide information on whether data, analytic code and/or other materials are accessible, and where and how they can be accessed.	Data availability statement
**20**	Funding sources	Provide the sources of funding and detail the role of the funders in the design, conduct and reporting of the study.	Funding information
**21**	Conflicts of interest	State any conflicts of interest and financial disclosures for all authors.	Conflict of interest statement

Supplementary Table S3: Bias Identification and Sensitivity Analysis Strategy Matrix

Dimension	Potential Risks & Concerns	Sensitivity Analysis Strategy	Rationale & Validation Logic
Inherent Data Source Biases	1. Proxy Prescriptions: In real-world reimbursement settings, medication may be collected by family members ("proxy prescription"), meaning the enrolled subject may not be the actual user.2. Endpoint Accuracy: MACE is a composite outcome. Relying solely on the first occurrence may overlook fatal events following non-fatal stroke/MI; cause-of-death coding in claims data has uncertainty.3. Unknown Adherence: Prescription records do not equate to actual medication intake.	1. Verified Medication Users: Analyze a subgroup with confirmed glycemic testing (e.g., HbA1c) within 14 days prior to the index prescription to exclude proxy users.2. Outcome Redefinition: (a) Reclassify all-cause death within 30 days of stroke/MI as CV death; (b) Analyze All-cause Mortality as a separate endpoint.3. Adherence Reinforcement: Censoring upon discontinuation/switching; restricted analysis to patients with continuous use >180 days (simulating a per-protocol set).	1. Data Quality Validation: Verify results in a high-quality subset to exclude false associations driven by "ghost patients".2. Specificity Verification: Assess the impact of cause-of-death misclassification and capture total survival benefits.3. Exposure Validation: Ensure the observed effect is derived from sufficient biological exposure duration.
Subgroup & Heterogeneity	1. Effect Modification: Treatment benefits may vary across demographic or clinical subgroups.2. Confounding by Severity: Claims data lack clinical severity markers (e.g., infarct size). Unmeasured confounding may differ between high-risk and low-risk groups.	1. Standard Stratification: Stratified analysis by age (<60 vs. ≥60), sex, baseline CVD history, and insulin use.2. Low-risk Population Analysis: Validation in a primary prevention cohort (excluding patients with baseline MI/Stroke).	1. Consistency Check: Evaluate robustness across clinical profiles and identify potential effect modifiers.2. Baseline Confounding Reduction: Validate benefits in a population with a "cleaner" disease background where unmeasured severity bias is minimized.
Model Assumptions	1. Selection Bias: Propensity Score Matching (PSM) may discard unmatched samples, introducing sampling bias.2. Model Dependency: Results might be sensitive to specific covariate selection or statistical modeling choices.	1. IPTW & Trimming: Use Inverse Probability Treatment Weighting (IPTW) to include all samples; apply truncated IPTW (trimming extreme weights <1% or >99%) to address positivity violations.2. Covariate Sensitivity: Systematically adding/removing covariates to construct alternative PS models.	1. Common Support Verification: Eliminate the influence of extreme weights and ensure comparability without sample loss.2. Robustness Check: Assess the marginal impact of model specification on effect estimates.
Unmeasured Confounding	1. Residual Confounding: Unmeasured factors may be unevenly distributed between groups, biasing results.2. Quantitative Uncertainty: Need to quantify how strong a bias must be to invalidate the conclusions.	1. Negative Control Outcomes: Analyze endpoints theoretically unrelated to the drug (e.g., fractures, head injuries) as falsification tests.2. E-value Calculation: Compute the E-value for the point estimate and lower confidence limit.	1. Falsification Test: A significant association with negative controls would suggest "Healthy User Bias" or systematic error.2. Threshold Quantification: Determine the minimum strength of association required for an unmeasured confounder to explain away the observed effect.

Supplementary Table S4: Demographic Characteristics, Concomitant Medications, and Medical History of Study Populations Before and After PS Matching

Demographic Characteristics	Unmatched population			Matched population		
	**Liraglutide （N=9038）**	**DPP4i （N=39353）**	**SMD**	**Liraglutide （N=8886）**	**DPP4i （N=8886）**	**SMD**
Sex			0.065			0.01
Male	4837 (53.5)	19786 (50.3)		4746 (53.4)	4790 (53.9)	
Female	4201 (46.5)	19567 (49.7)		4140 (46.6)	4096 (46.1)	
Age (years)*			0.285			0.009
Mean (Standard Deviation)	63.67 (8.6)	66.20 (9.1)		63.78 (8.6)	63.85 (8.6)	
Health Insurance Type*			0.587			0.01
Basic Medical Insurance for Urban and Rural Residents	980 (10.8)	4235 (10.8)		963 (10.8)	946 (10.6)	
Basic Medical Insurance for Employees	8058 (89.2)	35118 (89.2)		7923 (89.2)	7940 (89.4)	
Ethnicity			0.006			0.021
Han Chinese	8593 (95.1)	37470 (95.2)		8445 (95.0)	8484 (95.5)	
Minority Ethnic Groups	445 (4.9)	1883 (4.8)		441 (5.0)	402 (4.5)	
Marital Status			0.024			0.035
Married	5444 (60.2)	23312 (59.2)		5348 (60.2)	5300 (59.6)	
Divorced	100 (1.1)	396 (1.0)		99 (1.1)	79 (0.9)	
Widowed	51 (0.6)	230 (0.6)		51 (0.6)	43 (0.5)	
Unmarried	3435 (38.0)	15378 (39.1)		3380 (38.0)	3460 (38.9)	
Unspecified Marital Status	8 (0.1)	37 (0.1)		8 (0.1)	4 (0.0)	
Education Level*			0.112			0.016
Primary Education	362 (4.0)	2403 (6.1)		360 (4.1)	380 (4.3)	
Secondary Education	5836 (64.6)	25551 (64.9)		5750 (64.7)	5700 (64.1)	
Higher Education	1798 (19.9)	6784 (17.2)		1753 (19.7)	1787 (20.1)	
Unknown	1042 (11.5)	4615 (11.7)		1023 (11.5)	1019 (11.5)	
Occupation*			0.201			0.017

Demographic Characteristics	Unmatched population			Matched population		
	Liraglutide （N=9038）	DPP4i （N=39353）	SMD	Liraglutide （N=8886）	DPP4i （N=8886）	SMD
Civil Servant	100 (1.1)	306 (0.8)		98 (1.1)	100 (1.1)	
Farmer	429 (4.7)	1678 (4.3)		419 (4.7)	408 (4.6)	
Retiree	6423 (71.1)	31055 (78.9)		6354 (71.5)	6349 (71.4)	
Unemployed	306 (3.4)	1212 (3.1)		302 (3.4)	325 (3.7)	
Employed	1765 (19.5)	4994 (12.7)		1698 (19.1)	1692 (19.0)	
Other	15 (0.2)	108 (0.3)		15 (0.2)	12 (0.1)	
Year of First Use of the study drug						
2019	1738 (19.2)	11854 (30.1)	0.358	1734 (19.5)	1804 (20.3)	0.098
2020	1199 (13.3)	7207 (18.3)		1194 (13.4)	1276 (14.4)	
2021	1759 (19.5)	6672 (17.0)		1746 (19.6)	1445 (16.3)	
2022	1296 (14.3)	5206 (13.2)		1278 (14.4)	1384 (15.6)	
2023	1720 (19.0)	4984 (12.7)		1688 (18.8)	1621 (18.2)	
2024	1326 (14.7)	3430 (8.7)		1266 (14.2)	1356 (15.3)	
Interval from initial diabetes diagnosis to index date (days)			0.267			0.013
Mean (Standard Deviation)	838.2 (632.4)	676.9 (576.5)		833.1 (629.6)	831.0 (630.1)	
Clinical Characteristics						
CCI*			0.07			0.003
Mean (Standard Deviation)	4.96 (1.66)	4.84 (1.70)		4.95 (1.65)	4.94 (1.70)	
Concurrent liver disease*			0.157			0.013
Yes	1621 (17.9)	4848 (12.3)		1537 (17.3)	1493 (16.8)	
No	7417 (82.1)	34505 (87.7)		7349 (82.7)	7393 (83.2)	
Concurrent memory impairment (including dementia) *			0.059			<0.01
Yes	202 (2.2)	1255 (3.2)		200 (2.3)	200 (2.3)	
No	8836 (97.8)	38098 (96.8)		8686 (97.7)	8686 (97.7)	
Concurrent connective tissue disease			0.031			0.017
Yes	888 (9.8)	4240 (10.8)		876 (9.9)	922 (10.4)	
No	8150 (91.2)	35113 (89.2)		8010 (90.1)	7964 (89.6)	

Demographic Characteristics	Unmatched population			Matched population		
	**Liraglutide (N=9038)**	**DPP4i (N=39353)**	**SMD**	**Liraglutide (N=8886)**	**DPP4i (N=8886)**	**SMD**
Concurrent chronic pulmonary disease*			0.036			0.012
Yes	3643 (40.3)	16554 (42.1)		3588 (40.4)	3639 (41.0)	
No	5395 (59.7)	22799 (57.9)		5298 (59.6)	5247 (59.0)	
Concurrent cerebrovascular disease*			0.083			0.004
Yes	3720 (41.2)	17809 (45.3)		3666 (41.3)	3647 (41.0)	
No	5318 (58.8)	21544 (54.7)		5220 (58.7)	5239 (59.0)	
Hemiplegia			0.011			0.014
Yes	36 (0.4)	184 (0.5)		35 (0.4)	43 (0.5)	
No	9002 (99.6)	39169 (99.5)		8851 (99.6)	8843 (99.5)	
Concurrent renal disease			0.065			0.014
Yes	2086 (23.1)	8033 (20.4)		2038 (22.9)	2091 (23.5)	
No	6952 (76.9)	31320 (99.6)		6848 (77.1)	6795 (76.5)	
Concurrent heart disease			0.007			0.01
Yes	7982 (88.3)	34840 (88.5)		7861 (88.5)	7832 (88.1)	
No	1056 (11.7)	4513 (11.5)		1025 (11.5)	1054 (11.9)	
Concurrent dyslipidemia*			0.128			0.019
Yes	7379 (81.6)	30087 (76.5)		7238 (81.5)	7172 (80.7)	
No	1659 (18.4)	9266 (23.5)		1648 (18.5)	1714 (19.3)	
Concurrent peripheral vascular disease			0.087			0.014
Yes	5705 (63.1)	23165 (58.9)		5586 (62.9)	5525 (62.2)	
No	3333 (39.9)	16188 (41.1)		3300 (37.1)	3361 (37.8)	
Concurrent obesity*			0.255			0.016
Yes	485 (5.4)	528 (1.3)		387 (4.4)	359 (4.0)	
No	8553 (94.6)	38825 (98.7)		8499 (95.6)	8527 (96.0)	
Concurrent cholecystitis			0.008			0.012
Yes	273 (3.0)	1244 (3.2)		271 (3.0)	289 (3.3)	
No	8765 (97.0)	38109 (96.8)		8615 (97.0)	8597 (96.7)	
Complicated pancreatitis*			0.001			0.014

Demographic Characteristics	Unmatched population			Matched population		
	**Liraglutide （N=9038）**	**DPP4i （N=39353）**	**SMD**	**Liraglutide （N=8886）**	**DPP4i （N=8886）**	**SMD**
Yes	7 (0.1)	32 (0.1)		7 (0.1)	4 (0.0)	
No	9031 (99.9)	39321 (99.9)		8879 (99.9)	8882 (100.0)	
**Medical History**						
Previous use of alpha-glucosidase inhibitors*			0.088			0.005
Yes	4383 (48.5)	20816 (52.9)		4330 (48.7)	4309 (48.5)	
No	4655 (51.5)	18537 (47.1)		4556 (51.3)	4577 (51.5)	
Previous use of glinide hypoglycemic agents*			0.036			0.007
Yes	568 (6.3)	2833 (7.2)		562 (6.3)	578 (6.5)	
No	8470 (93.7)	36520 (92.8)		8324 (93.7)	8308 (93.5)	
Previous use of GKA hypoglycemic agents			0.023			<0.01
Yes	13 (0.1)	27 (0.1)		13 (0.1)	13 (0.1)	
No	9025 (99.9)	39326 (99.9)		8873 (99.9)	8873 (99.9)	
Previous use of biguanide hypoglycemic agents*			0.089			0.019
Yes	5761 (63.7)	23375 (59.4)		5644 (63.5)	5563 (62.6)	
No	3277 (36.3)	15978 (40.6)		3242 (36.5)	3323 (37.4)	
Previous use of peripheral vasodilators			0.02			0.007
Yes	683 (7.6)	2767 (7.0)		667 (7.5)	650 (7.3)	
No	8355 (92.4)	36586 (93.0)		83219 (92.5)	8236 (92.7)	
Previous use of thiazolidinedione hypoglycemic agents*			0.051			0.004
Yes	903 (10.0)	3349 (8.5)		881 (9.9)	871 (9.8)	
No	8135 (90.0)	36004 (91.5)		8005 (90.0)	8015 (90.2)	
Previous use of cardiac medications			0.068			0.01
Yes	4689 (51.9)	19073 (48.5)		4615 (51.9)	4659 (52.4)	
No	4349 (48.1)	20280 (51.5)		4271 (48.1)	4227 (47.6)	
Previous use of cardiac glycosides*			0.028			0.017
Yes	106 (1.2)	587 (1.5)		106 (1.2)	123 (1.4)	
No	8932 (98.8)	38766 (98.5)		8780 (98.8)	8763 (98.6)	

Demographic Characteristics	Unmatched population Liraglutide (N=9038)	Unmatched population DPP4i (N=39353)	Unmatched population SMD	Matched population Liraglutide (N=8886)	Matched population DPP4i (N=8886)	Matched population SMD
Previous use of second-generation sulfonylurea derivatives*			0.142			0.013
Yes	1958 (21.7)	10924 (27.8)		1940 (21.8)	1894 (21.3)	
No	7080 (78.3)	28429 (72.2)		6946 (78.2)	6992 (78.7)	
Previous use of insulin*			0.416			0.001
Yes	5708 (63.2)	16859 (42.8)		5564 (62.6)	5558 (62.5)	
No	3330 (36.8)	22494 (57.2)		3322 (37.4)	3328 (37.5)	
Previous use of short-acting GLP-1 agonists*			0.038			0.017
Yes	9 (0.1)	4 (0.0)		8 (0.1)	4 (0.0)	
No	9029 (99.9)	39349 (100.0)		8878 (99.9)	8882 (100.0)	
Previous use of long-acting GLP-1 agonists*			0.093			0.014
Yes	78 (0.9)	75 (0.2)		63 (0.7)	53 (0.6)	
No	8960 (99.1)	39278 (99.8)		8823 (99.3)	8833 (99.4)	
Previous use of PPAR agonists*			0.032			0.009
Yes	8 (0.1)	6 (0.0)		7 (0.1)	5 (0.1)	
No	9030 (99.9)	39347 (100.0)		8879 (99.9)	8881 (100.0)	
Previous use of SGLT-2 inhibitors*			0.088			0.011
Yes	300 (3.3)	755 (1.9)		286 (3.2)	269 (3.0)	
No	8738 (96.7)	38598 (98.1)		8600 (96.8)	8617 (97.0)	
Previous use of antihypertensive drugs*			0.01			0.002
Yes	802 (8.9)	3381 (8.6)		795 (8.9)	800 (9.0)	
No	8236 (91.1)	35972 (91.4)		8091 (91.1)	8086 (91.0)	
Previous use of ACE inhibitors			0.013			0.007
Yes	1105 (12.2)	4648 (11.8)		1088 (12.2)	1069 (12.0)	
No	7933 (87.8)	34705 (88.2)		7798 (87.8)	7817 (88.0)	
Previous use of ARBs*			0.105			0.007
Yes	4827 (53.4)	18956 (48.2)		4727 (53.2)	4698 (52.9)	
No	4211 (46.6)	20397 (51.8)		4159 (46.8)	4188 (47.1)	
Previous use of ARNI drugs			0.072			0.007

Demographic Characteristics	Unmatched population			Matched population		
	**Liraglutide （N=9038）**	**DPP4i （N=39353）**	**SMD**	**Liraglutide （N=8886）**	**DPP4i （N=8886）**	**SMD**
Yes	276 (3.1)	760 (1.9)		267 (3.0)	277 (3.1)	
No	8762 (96.9)	38593 (98.1)		8619 (97.0)	8609 (96.9)	
Previous use of lipid-lowering drugs*			0.109			0.011
Yes	1838 (20.3)	6342 (16.1)		1784 (20.1)	1745 (19.6)	
No	7200 (79.7)	33011 (83.9)		7102 (79.9)	7141 (80.4)	
Previous use of lipid-lowering traditional Chinese medicine			0.003			0.006
Yes	4 (0.0)	15 (0.0)		4 (0.0)	3 (0.0)	
No	9034 (100.0)	39338 (100.0)		8882 (100.0)	8883 (100.0)	
Previous use of diabetic neuropathy drugs*			0.078			0.003
Yes	2923 (32.3)	11310 (28.7)		2857 (32.2)	2844 (32.0)	
No	6115 (67.7)	28043 (71.3)		6029 (67.8)	6042 (68.0)	
Previous use of antithrombotic drugs*			0.078			0.008
Yes	6648 (73.6)	27575 (70.1)		6543 (73.6)	6512 (73.3)	
No	2390 (26.4)	11778 (29.9)		2343 (26.4)	2374 (26.7)	
Previous use of vascular protectants			0.055			0.015
Yes	913 (10.1)	3342 (8.5)		898 (10.1)	859 (9.7)	
No	8125 (89.9)	36011 (91.5)		7988 (89.9)	8027 (91.5)	
Previous use of CCB antihypertensive drugs*			0.03			0.005
Yes	5242 (58.0)	22243 (56.5)		5156 (58.0)	5135 (57.8)	
No	3796 (42.0)	17110 (43.5)		3730 (42.0)	3751 (42.2)	
Previous use of diuretic antihypertensive drugs			0.045			0.004
Yes	1278 (14.1)	4961 (12.6)		1257 (14.1)	1269 (14.3)	
No	7760 (85.9)	34392 (87.4)		7629 (85.9)	7617 (87.4)	
Previous use of statin lipid-lowering drugs*			0.125			0.009
Yes	6832 (75.6)	27571 (70.1)		6713 (75.5)	6678 (75.2)	
No	2206 (24.4)	11782 (29.9)		2173 (24.5)	2208 (24.8)	
Previous use of beta-blocker antihypertensive drugs*			0.128			0.015

Demographic Characteristics	Unmatched population Liraglutide (N=9038)	Unmatched population DPP4i (N=39353)	Unmatched population SMD	Matched population Liraglutide (N=8886)	Matched population DPP4i (N=8886)	Matched population SMD
Yes	4326 (47.9)	16340 (41.5)		4238 (47.7)	4303 (48.4)	
No	4712 (52.1)	23013 (48.5)		4648 (52.3)	4583 (51.6)	
Visits to secondary or higher-level hospitals during baseline period*			0.278			0.006
Yes	1156 (12.8)	9202 (23.4)		1155 (13.0)	1172 (13.2)	
No	7882 (87.2)	30151 (76.6)		7731 (87.0)	7714 (86.8)	
Number of hospitalizations during baseline period*			0.273			0.025
Mean (SD)	0.51 (0.7)	0.33 (0.7)		0.50 (0.7)	0.52 (0.8)	
Number of outpatient visits during baseline period*			0.036			0.013
Mean (SD)	14.39 (8.9)	14.71 (9.3)		14.42 (8.9)	14.54 (8.8)	

*Variables used for matching in the PS model.
